# Changing epidemiology of methicillin-resistant *Staphylococcus aureus* in a low endemicity area—new challenges for MRSA control

**DOI:** 10.1007/s10096-020-03824-9

**Published:** 2020-01-27

**Authors:** Jenna Junnila, Tiina Hirvioja, Esa Rintala, Kari Auranen, Kaisu Rantakokko-Jalava, Jaakko Silvola, Laura Lindholm, Kirsi Gröndahl-Yli-Hannuksela, Harri Marttila, Jaana Vuopio

**Affiliations:** 1 Institute of Biomedicine, University of Turku, Turku, Finland; 2 Department of Hospital Hygiene & Infection Control, Turku University Hospital, Turku, Finland; 3 Department of Mathematics and Statistics, and Department of Clinical Medicine, University of Turku, Turku, Finland; 4 Clinical Microbiology Laboratory, Turku University Hospital, Turku, Finland; 5 Department of Health Security, Finnish Institute for Health and Welfare, Helsinki, Finland

**Keywords:** MRSA, Community-associated, Infection control, Family cluster, Epidemiology, *spa* type

## Abstract

The incidence of methicillin-resistant *Staphylococcus aureus* (MRSA) has increased sharply in Hospital District of Southwest Finland (HD). To understand reasons behind this, a retrospective, population-based study covering 10 years was conducted. All new 983 MRSA cases in HD from January 2007 to December 2016 were analysed. Several data sources were used to gather background information on the cases. MRSA cases were classified as healthcare-associated (HA-MRSA), community-associated (CA-MRSA), and livestock contact was determined (livestock-associated MRSA, LA-MRSA). *Spa* typing was performed to all available strains. The incidence of MRSA doubled from 12.4 to 24.9 cases/100000 persons/year. The proportion of clinical infections increased from 25 to 32% in the 5-year periods, respectively, (*p* < 0.05). The median age decreased from 61 years in 2007 to 30 years in 2016. HA-MRSA accounted for 68% of all cases, of which 32% associated with 26 healthcare outbreaks. The proportion of CA-MRSA cases increased from 13% in 2007 to 43% in 2016. Of CA-MRSA cases, 43% were among family clusters, 32% in immigrants and 4% were LA-MRSA. The Gini-Simpson diversity index for *spa* types increased from 0.86 to 0.95 from the first to the second 5-year period. The proportion of a predominant strain t172 decreased from 43% in 2009 to 7% in 2016. The rise in the proportion of CA-MRSA, the switch to younger age groups, the complexity of possible transmission routes and the growing *spa*-type diversity characterize our current MRSA landscape. This creates challenges for targeted infection control measures, demanding further studies.

## Introduction

High and increasing rates of methicillin-resistant *Staphylococcus aureus* (MRSA) infection remain a global concern [1, 2]. Nevertheless, Finland has been a country with relatively low MRSA incidence. Since 2007, there has been a significant increase in the number of MRSA cases in one of the hospital districts with previously low incidence in Southwest Finland [3]. We conducted a retrospective, population-based study to identify reasons behind this emergence. An epidemiological survey together with strain-type analysis for all 983 MRSA cases from January 2007 through December 2016 was performed. In Finland, MRSA findings from both clinical infections and carriers are recorded and investigated, which allowed us to study the complexity of MRSA acquisitions in this setting.

## Materials and methods

### Background and data collection

The hospital district of Southwest Finland (HD) is a public joint municipal healthcare authority, consisting of 28 member municipalities with a total of 478,500 residents. The population of HD represents 8.7% of the population in Finland (5.5 million).

All clinical microbiology laboratories in Finland notify new MRSA findings from both clinical and screening samples to the National Infectious Disease Register (NIDR) maintained by the Finnish institute for health and welfare (THL). The Turku University Hospital clinical microbiology laboratory notifies MRSA cases also to a regional Register of Hospital Infections and Carriers of Resistant Bacteria (SAI register, SAI®, Neotide Oy, Vaasa, Finland) kept by HD. An individual with a positive MRSA finding (MRSA case) is notified to SAI only once irrespective of the number of positive cultures after the first notification. In each new MRSA case, hospital contacts and risk factors for transmission are tracked by an infection control nurse. The patient records of hospital contacts are tagged with screening alert for future admissions.

All MRSA cases in HD from January 2007 through December 2016 were included in the analysis. Predisposing factors for MRSA carriage were obtained from the SAI register, patient records and a patient questionnaire designed for identifying community-associated MRSA [4]. The incidence rate of MRSA was obtained from the NIDR. Data on hospital care were derived from the national Care Register for Health Care (HILMO) kept by THL.

MRSA cases in healthcare workers (HCWs), infants under 28 days of age, and patients who were hospitalized or who had stayed in a nursing home within the previous 2 years in Finland, or 1 year abroad, were defined as healthcare-associated MRSA (HA-MRSA). Cases without such criteria were defined as community-associated MRSA (CA-MRSA) if MRSA was isolated at outpatient care or within the first 2 days of hospital care. If MRSA was isolated later during hospital care, the case was defined as HA-MRSA. Clinical samples were defined as diagnostic bacterial culture from the site of infection whereas screening samples were taken from asymptomatic individuals according to screening protocols. Each case was defined either “clinical infection” or “asymptomatic carrier” based on the sample type from which MRSA was first identified.

Healthcare outbreaks were defined as two or more MRSA cases in patients who had shared a room or washing facility in a healthcare ward and MRSA cases in HCWs working in the same ward. Family clusters were defined as two or more MRSA cases living in the same household. For each outbreak and cluster, the index case was defined as the first identified case leading to outbreak or cluster investigation. Livestock-associated MRSA (LA-MRSA) cases were defined as MRSA cases in pig farmers, their household members or employees working at the farm.

In addition to standard precautions, infection control (IC) practices in HD to prevent MRSA outbreaks include targeted screening of patients attending hospital care, barrier precautions and isolation rooms in the care of MRSA carriers, as well as MRSA cohort units in long-term care. At admission to hospital, screening samples are routinely taken from patients who have stayed in a hospital or nursing home abroad within previous year prior to hospitalisation, and from asylum seekers (targeted screening since 2015). When a new MRSA case has been identified in hospital or long-term care, all exposed patients, determined by a shared room or toilet or washing facility within the previous year, are traced and screened promptly or at next hospital admission. In Finland, screening is also performed in patients exposed in a hospital or nursing home with an MRSA outbreak. HCWs are screened in case of an unresolved MRSA outbreak in a ward or after working in healthcare facilities abroad within the previous year. Household contacts of MRSA carriers are screened before decolonisation of the carrier and at admission to hospital. MRSA cultures for screening are taken from the anterior nares, throat, armpits, groins, perineum, and skin lesions.

### Microbiological methods

Clinical samples were inoculated, and *S. aureus* identified according to standard methods, including rapid coagulase tests, Vitek2 and since 2011, Maldi-TOF MS. Methods for MRSA surveillance varied during the study period but included use of chromogenic MRSA-agars (since 2007) and enrichment broth (since 2008). Cefoxitin was used for selection of MRSA in both enrichment and antimicrobial susceptibility testing by disk diffusion according to CLSI and since 2011 the EUCAST methodology. The presence of *mecA* (and since 2014, *mecC*) was confirmed by a molecular method throughout the study period.

Finnish clinical microbiology laboratories send all new MRSA isolates to THL for strain typing. The standard methods for MRSA typing were pulsed field gel electrophoresis (PFGE) in 2007–2008, and *spa* typing since 2009 to date. *Spa* typing was performed as described elsewhere [5, 6] for all available strains isolated in 2007–2008. The *spa* sequence was analysed by using the Ridom StaphType™ software (Ridom GmbH, Würzburg, Germany).

### Statistical methods

Trends in median age and in the proportion of HA-MRSA/CA-MRSA cases were studied using median regression and the chi-squared test for trend in proportions, respectively. The Gini-Simpson diversity index of *spa* types was estimated for time periods 2007–2011 and 2012–2016 (the former and the latter 5-year period). In addition to the overall diversity, diversity was calculated separately for HA-MRSA and CA-MRSA cases, and similarly for clinical and screening samples. Demographic variables of the MRSA carrier population were compared between the two time periods using chi-squared test or Fisher’s exact test. For each *spa* type carried by at least 10 persons, its association with the HA-MRSA/CA-MRSA and clinical infection/asymptomatic carrier case status was evaluated using chi-squared test or Fisher’s exact test. Data analyses were performed with IBM SPSS Version 25 for Windows (IBM Corp., Armonk, NY) and with R for Windows, version 3.6.0.

## Results

**Fig. 1:**
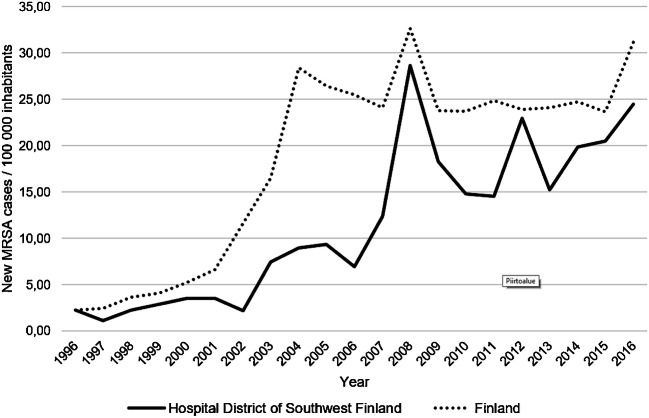
Incidence (per 100,000 inhabitants) of methicillin-resistant *Staphylococcus aureus* (MRSA) in Hospital District of Southwest Finland and in the whole country in 1996–2016. Source: Finnish Institute for Health and Welfare

A total of 983 new MRSA cases were identified in HD during 2007–2016 (range 62 to 134 cases/year). The incidence of MRSA increased 2.0-fold from 12.4 to 24.9 cases/100000 persons/year during the study period. In the whole country, the incidence of MRSA increased 1.3-fold from 24.1 to 31.2 cases/100000 persons/year during the same time period (Fig. 1).

**Fig. 2:**
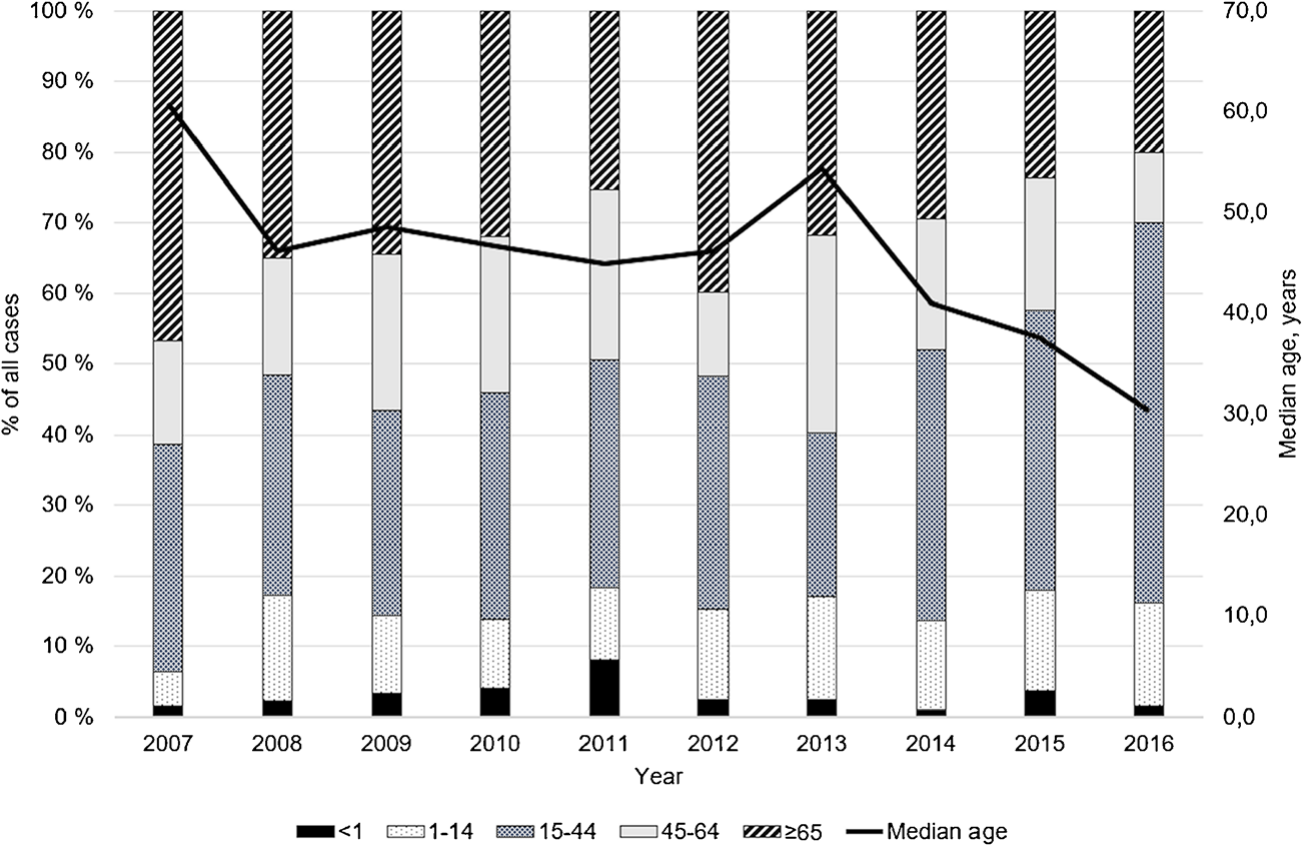
Age groups and median age of MRSA cases in 2007–2016 in Hospital District of Southwest Finland. The columns show yearly proportions (%) of age groups (age in years), and the line indicates the yearly median age of MRSA cases

- The median age of MRSA cases was 43 years (range 0–103 years). The median age decreased from 61 years in 2007 to 30 years in 2016, corresponding to a 2.6-year decline in the median age per calendar year (95% CI 1.5–3.3). The proportion of cases ≥ 65 years of age decreased (range 20–47%/year) but the proportion of cases under 15 years of age remained relatively low (range 6–18%/year) throughout the study period (Fig. 2). The gender distribution remained unaltered between the two study periods (Table 1).

**Table 1:** Demographics, epidemiological classification or predisposing risk factors for MRSA cases in Hospital District of Southwest Finland in the time periods 2007–2011 and 2012–2016

	Time period		
	2007–2011	2012–2016	
	*N* = 445	*N* = 538	*p*
Age (years)	Median 48.5 Range 0–97	Median 39.1 Range 0–103	< 0.001 ^a)^
Male, *n* (%)	202 (45.4)	243 (45.2)	0.944
Clinical MRSA infection, *n* (%)	110 (24.7)	170 (31.6)	0.017
Healthcare-associated case (HA), *n* (%)	324 (72.8)	349 (64.9)	0.008
Healthcare outbreak associated case, *n* (%)	121 (27.2)	96 (17.8)	< 0.001
Stay at a long-term care facility, *n* (%)	95 (21.3)	81 (15.1)	0.010
Hospital care abroad within 2 years, *n* (%)	76 (17.1)	110 (20.4)	0.180
Healthcare worker, *n* (%)	40 (9.0)	29 (5.4)	0.028
Community-associated case (CA), *n* (%)	121 (27.2)	189 (35.1)	0.008
Other identified risk groups:			
Family cluster associated case, n (%)	169 (38.0)	135 (25.1)	< 0.001
Asylum seeker or refugee, n (%)	11 (2.3)	75 (13.9)	< 0.001
History of intravenous drug abuse, n (%)	4 (0.9)	7 (1.3)	b
Livestock-associated case, n (%)	2 (0.4)	20 (3.7)	b

^a^Median regression analysis, ^b^Not calculated

Of all MRSA cases, 28% (280/983, range 18–38% /year) were diagnosed from clinical samples taken from the site of infection. The proportion of clinical infections was higher in the latter 5-year period as compared with the first 5-year period (25% vs. 32%, *p* < 0.05) (Table 1).

**Fig. 3:**
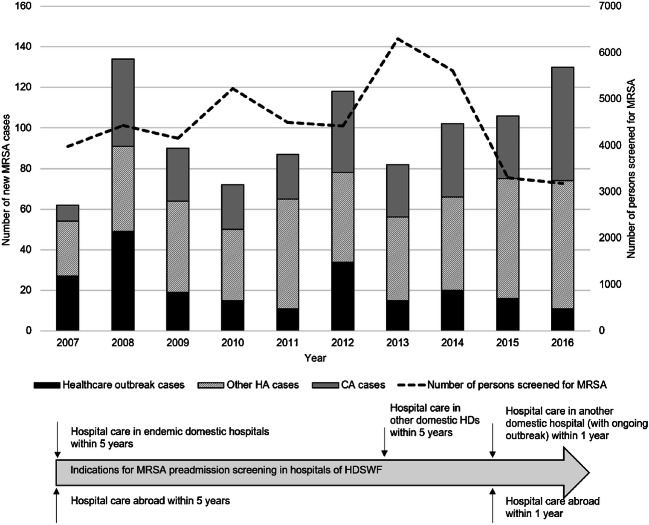
MRSA cases and MRSA screening in Hospital District of Southwest Finland in 2007–2016. The graph columns show the number of MRSA cases by year divided in community-associated MRSA (CA-MRSA), solitary healthcare-associated MRSA (HA-MRSA) and outbreak-associated HA-MRSA cases. The line indicates the number of persons screened yearly with its scale on the right side of the graph. The timeline below the graph shows changes in the indications for MRSA screening in the Hospital District of Southwest Finland

Although the indications for screening became more focused, the number of MRSA cases increased during the last few study years (Fig. 3).

### HA-MRSA and outbreaks in hospitals and long-term care facilities

A total of 673 (68%) MRSA cases were classified as HA-MRSA (Fig. 3). The proportion of HA-MRSA of all MRSA cases was 87% (54/62) in 2007 and 57% (74/130) in 2016, corresponding to a 7.0% decrease per calendar year (95% CI 2.6–11.3) in the odds of HA-MRSA. The median age of HA-MRSA cases was 58 years (range 0–103 years). Clinical infections accounted for 27% (182/673) of the HA-MRSA cases.

Of all HA-MRSA cases, long-term care facility residents accounted for 26% (176/673) and infants under 28 days of age for 1% (9/673). The remaining 63% (419/673) had been in hospital care, of which 32% (134/419) abroad. One-third (32%, 217/673) had an epidemiological link to an outbreak in a healthcare facility**.** In all, we identified 26 healthcare outbreaks, the median number of cases per outbreak being five (range 2–32). Five of the healthcare outbreaks occurred in the University Hospital, seven in municipal health centre wards and 14 in long-term care facilities. There were thirteen outbreaks in both 2007–2011 and 2012–2016, although there was a decrease in the number of cases linked to the outbreaks (121 and 96 cases, respectively).

HCWs accounted for 10% (69/673) of the HA-MRSA cases, and their proportion of all MRSA cases decreased from 9% (40/445) in the first 5-year period to 5.4% (29/538) in the latter (Table 1).

### CA-MRSA

A total of 310 (32%) MRSA cases were classified as CA-MRSA. The proportion of CA-MRSA of all MRSA cases was 13% (8/62) in 2007 and 43% (56/130) in 2016, corresponding to a 7.5% increase per calendar year (95% CI 2.6–12.7) in the odds of CA-MRSA (Fig. 3). The median age of CA-MRSA cases was 28 years (range 0–92). Clinical infections accounted for 32% (98/310) of CA-MRSA cases with no statistically significant difference in the proportion as compared with HA-MRSA. Half (49%, 152/310) of the CA-MRSA cases were members of family clusters. Immigrants, refugees and residents of foreign countries accounted for one-third of the CA-MRSA cases (32%, 99/310). A livestock contact was identified in 4% (13/310) of the cases. There is overlapping in the previously mentioned groups as there were immigrants and livestock farmers also in family clusters.

### MRSA in family clusters

We identified 129 MRSA family clusters. In 4 clusters, the index cases were detected before 2007, and in 4 family clusters, the household size was unknown. Thus, 304 MRSA cases in 121 family clusters were included in the analysis. The mean household size in family clusters was 3.2 persons (median 3, range 2–8). Of the 121 index cases, 70% (85/121) were HA-MRSA and 30% (36/121) CA-MRSA. Of the secondary household cases, 37% (67/183) were HA-MRSA and 63% (116/183) CA-MRSA. Table 1 shows the number of MRSA cases in family clusters in the two 5-year study periods.

### MRSA in immigrants

We identified a total of 213 (22%, 213/983) MRSA cases among the non-native/foreign-born population in HD, including asylum seekers and refugees, other immigrants and residents of foreign countries. Of those, 15% (31/213) were detected by clinical samples. Refugees accounted for 86 cases, of which 11 cases were detected during 2007–2011, and 76 cases in 2012–2016. Other immigrants accounted for 11% (113/983) of the cases, of which 25 and 88 cases were identified in the first and in the second half of the study period, respectively. MRSA was identified in six residents of foreign countries and in eight foreign healthcare exchange students.

### Livestock-associated MRSA cases

Altogether 22 (2%, 22/983) livestock-associated MRSA (LA-MRSA) cases were detected, the first ones in 2010. MRSA findings were detected from screening samples in 77% (17/22) of the cases, and from clinical infection sites in 23% (5/22) of the cases. Nine cases had also a healthcare association.

### Intravenous drug abuse

We identified 11 cases (1%, 11/983) with a documented history of intravenous drug abuse either previously or presently. Their proportions of all cases in the two 5-year periods were 0.9% and 1.3%, respectively (Table 1).

### *Spa*-type diversity

**Fig. 4:**
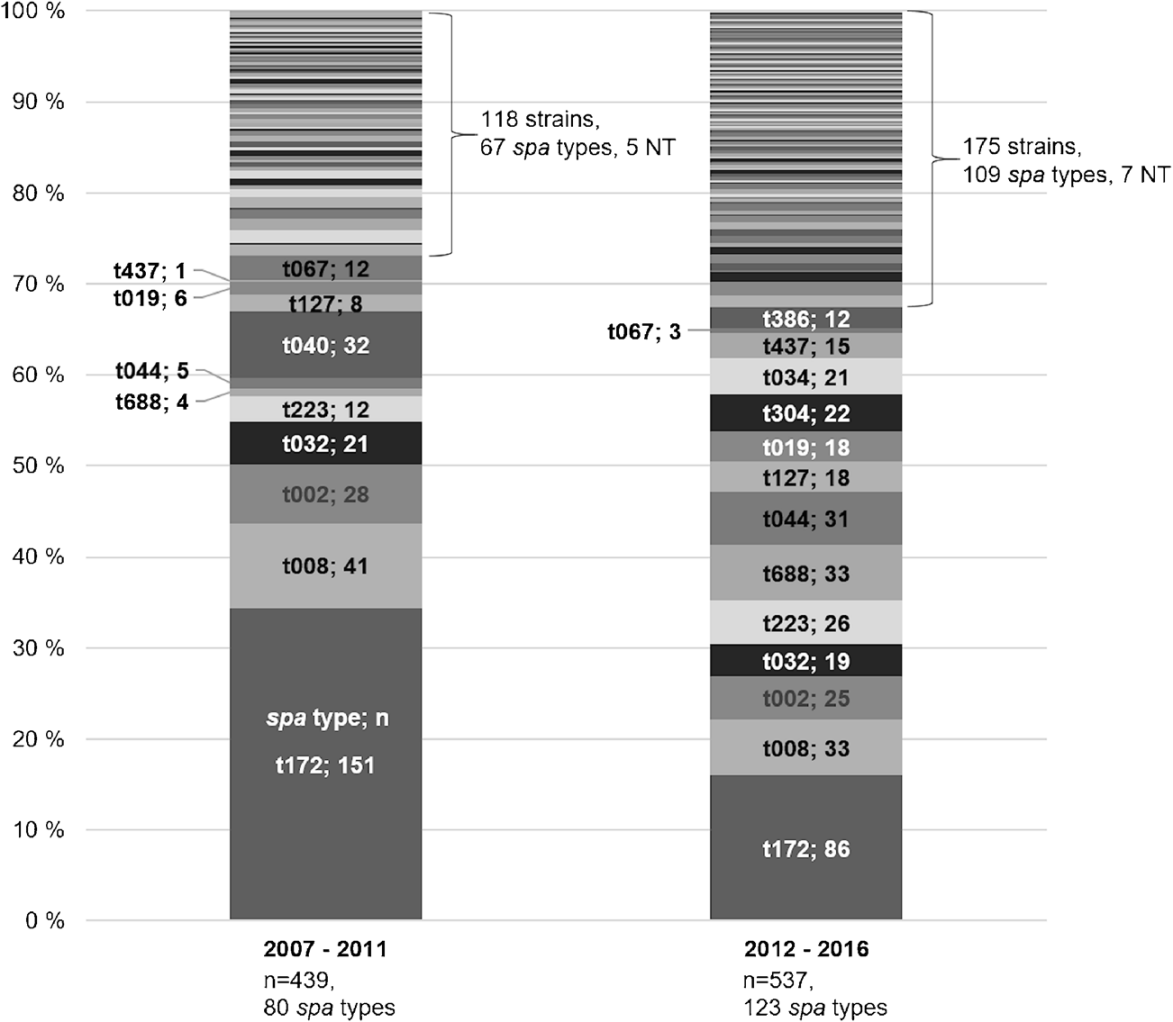
The *spa* types of altogether 976 MRSA strains in Hospital District of Southwest Finland in 2007–2011 and in 2012–2016. Each stripe indicates one *spa* type, and the height of the stripe represents the proportion of the type in each time period. The fifteen most common spa types (identified in at least 10 cases) are listed by type name, following by the number of cases. NT = non-typeable

*Spa* typing was performed on 99.3% (976/983) of the MRSA strains. Seven strains (0.7%) from years 2007 and 2008 were not available for typing. Altogether 173 different *spa* types were identified while 12 (1%, 12/976) strains remained non-typeable (NT). Fifteen *spa* types were identified from ≥ 10 cases, representing 70% of the strains, 60 *spa* types from 2 to 9 cases (19%), and 98 *spa* types were sporadic (10%). The most common *spa* types were t172 (24%, 237/976), t008 (8%, 74/976) and t002 (5%, 54/976). *Spa* type t172 was predominant throughout the study period, but its proportion first increased from 18% in 2007 to 43% in 2009, then decreased steadily down to 7% in 2016. The Gini-Simpson diversity index increased from 0.86 (95% CI 0.82–0.88) to 0.95 (95% CI 0.94–0.96) from the first to the second half of the study period (Fig. 4).

The *spa*-type diversity was similar between HA- and CA-MRSA groups (Gini-Simpson index 0.91 [95% CI 0.90–0.93] and 0.93 [95% CI 0.90–0.94], respectively), as well as between clinical infections and asymptomatic carriage groups (0.93 [95% CI 0.90–0.94] and 0.92 [95% CI 0.90–0.93], respectively). *Spa* types t032, t040, t688 and t067 were associated with HA-MRSA (*p* < 0.005), whereas *spa* types t002, t019, t034, t044 and t223 associated with CA-MRSA (*p* < 0.04). *Spa* types associated with clinical infections were t032 (*p* < 0.001), t044 and t127 (*p* < 0.05). *Spa* type t172 predominated in both HA-MRSA (25%, 170/673) and CA-MRSA (22%, 67/310) groups, as well as in healthcare outbreaks (33%, 71/217) and in family clusters (33%, 99/304), but not in the immigrant group (4%, 8/202), where the most common *spa* types were t223 and t044 (each 9%, 19/202). The LA-MRSA *spa* types were t034 (68%, 15/22), t2741 (14%, 3/22), t108 (9%, 2/22), t1250 and t032 (each 4.5%, 1/22).

## Discussion

In this study, we document an increase in the proportion of CA-MRSA in our hospital district (HD). Simultaneously, the boundaries between epidemiologically defined CA- or HA-MRSA cases have become increasingly blurry, indicated by the observed overlap of *spa* types between the different MRSA groups. This complicates planning of preventive actions against spreading of MRSA, especially in the community setting.

The incidence of MRSA doubled from 2007 to 2016 in our HD, still remaining below the national average level. Most of the increase is due to CA-MRSA. Previously, the incidence of CA-MRSA isolations had tripled in Finland in 1997–2006, but proportion of CA-MRSA decreased as the total amount of MRSA cases eightfolded [4, 7]. Reflecting the rise of CA-MRSA, there was a marked shift towards younger age groups, especially young adults. Comparably, in Sweden, the median age of all MRSA cases was 30 years [8], and in Norway, an increase in MRSA infections of persons under 70 years has been observed concurrently with an increase in CA-MRSA [9].

The *spa*-type diversity increased significantly in addition to the substantial decline of a previously dominant strain type t172. Previously, t172 was associated to CA-MRSA [4], but in our data, it was predominant also in HA-MRSA. Recently, increases in the diversity of MRSA *spa* types have been reported in other countries of low MRSA incidence [8, 10, 11]. Given that there are major geographical variations among prevalent *spa* types, the increased diversity is, at least partly, indicative of human migration and travelling [12, 13]. The receding or clonal replacement of predominant strains is also a known feature for MRSA [14, 15].

Clinical infections accounted for only 28% of all cases in our study. However, the proportion was on the rise in the latter period of the study. The prominence of asymptomatic carriers is common where an active “search-and-destroy” screening policy is exploited [8, 16]. The screening policy in Finland, as in other Nordic countries and in the Netherlands, aims to contain and prevent MRSA outbreaks, which seems to be effective in hospital settings [17–20]. In a country where screening is routine, it is often difficult to establish whether trends in MRSA incidence are true or due to modifications in screening policies. During our study period, the screening policy underwent some changes, resulting in a notable reduction in the annual number of screened individuals (Fig. 3). At the same time, the number of new MRSA cases increased, suggesting effectiveness of targeted screening (e.g. refugees) and a possible true increase of MRSA in the population. The increase in clinical MRSA infections supports the latter. In a Norwegian study, a time series analysis revealed an actual increase in MRSA incidence irrespective of the screening policies or the amount of *S. aureus* findings [21].

There was a declining trend in the proportion of HA-MRSA and the outbreaks in healthcare facilities were smaller in the latter period of the study. In 2007, 87% of MRSA cases were associated with healthcare, whereas in 2016 the proportion was only 57%. After large MRSA outbreaks in hospitals and long-term care facilities in the 1990’s [22], the frequency of healthcare-associated outbreaks and the number of affected patients per outbreak have declined in our HD. There were either single HA-MRSA cases or minor clusters in healthcare facilities, but no major outbreaks in the latter period of the study. In addition, the role of long-term care facilities in MRSA epidemiology has diminished. In 2001, MRSA isolates from long-term care facilities alone accounted for more than half of the MRSA cases in Finland [23], whereas in the latter period of our study, long-term care patients accounted only for 15% of the cases. Enhanced infection control measures such as use of isolation rooms or cohorts, screening of contact patients and enhanced hand hygiene have contributed to the control of MRSA epidemics in healthcare facilities [22].

MRSA findings in HCWs differed significantly between the two study periods. In the first period, HCWs were screened in uncontained hospital outbreaks, but in the latter period, in absence of hospital outbreaks, the need for screening diminished and HCWs were mainly screened only when having a history of work at hospitals abroad.

Intravenous drug users were not routinely screened for MRSA, and only those with a well-documented history of drug abuse in patient records were regarded as intravenous drug users. Thus, our number of MRSA findings in intravenous drug users is not conclusive.

A prominent MRSA group appeared to be household members of MRSA carriers. Our screening practice including household members of MRSA carriers proved very effective as altogether 19% (183/983) of MRSA cases were detected that way. A high risk of MRSA transmission between household members has been noted earlier [24, 25]. In a recent Norwegian study, it was estimated that up to 49% of new MRSA colonisation events were among household contacts of MRSA carriers [26]. Our results as well as findings from other low-prevalence countries support the policy of targeted screening of household members of MRSA carriers prior to or at hospital admission. Transmission of MRSA within exposed households is a complex interplay of colonized household members, pets and the environment, where the key determinants seem to be proximity, seasonality and hygiene practices [27, 28]. Our knowledge on effective and best practical measures to decrease the colonisation pressure of MRSA carriers, and their household environments is still at current limited.

Interestingly, a significant proportion of MRSA-positive household members of an index case in family clusters also possessed a contact to healthcare (and could, thus, be classified as HA-MRSA). A detailed analysis on the *spa* types, and furthermore, whole genome sequencing (WGS) of the family cluster-associated isolates could shed light on transmission routes.

The sharp increase in the numbers of refugees and asylum seekers in Europe in 2015 affected also Finland. Compared with 2014, the number of refugees ninefolded in Finland in 2015 yet declining sharply thereafter. The proportion of population from foreign origin in our HD was 4% in 2007 and 7% in 2016 [29]. Previous studies have reported MRSA prevalence rates of 21% in refugees in Finland [30], and up to 10 % in screening cultures and 19% in clinical cultures in the Netherlands [31]. In Norway, an increasing trend of CA-MRSA incidence due to immigration was reported [9]. In our HD, the shift of MRSA to younger age groups and the increase in *spa*-type diversity most likely reflect similar movements in the population.

Since 2010, pig farmers and their families along with farm workers emerged as a distinct but a relatively small group within our MRSA carrier population. Cases associated with pig farming (LA-MRSA) were mostly found in the last 2 years of the study. In comparison with Denmark and the Netherlands in Finland, the human LA-MRSA situation remained still moderate during our study years [32, 33]. This holds also for the municipalities of our HD where the majority of Finnish pig farms are located. Of the *spa* types among the LA-MRSA cases, 21/22 (95%) have previously been linked to livestock. *Spa* types t034, t108 and t1250 have been linked to livestock in Europe [34], and t2741 has been detected as a new dominant and highly adhesive strain in Finnish slaughtering pigs [35, 36]. Type t032 (1/22) is likely of human origin despite the livestock contact of the case.

Our study shows that the often-used epidemiological classification into HA-MRSA, CA-MRSA and LA- MRSA is too rigid as MRSA transmission may occur through multiple and interplaying sources. For example, a part of HA-MRSA cases in our study may in fact have originated from the community because, by definition, we allowed a rather long period (up to 2 years) between recorded hospital care period and the subsequent MRSA finding. Furthermore, although we did find some differences in the *spa*-type distribution between the HA- and CA-MRSA groups, those differences could have multiple explanations, strains originating from abroad being the most likely cause. Moreover, the most prevalent *spa* type t172 in our HD has previously been associated with CA-MRSA [4], but this could not be shown in our current study. Other researchers have also noted a significant overlap of identical clones across these three MRSA-groups [37].

The strength of this study was the use of multiple data sources, including epidemiological data, patient interviews, patient records and universal *spa* typing of MRSA strains for a 10-year period. Detailed review of data files of each MRSA case could be performed which improves the quality of analysis. Restricting the study to one hospital district alone means that the similar practices over the long study period could be employed, and any changes were traceable.

In conclusion, HA-, CA- and LA-MRSA strains intermingle in community and healthcare, making analyses of exact transmission routes difficult. The prevention of the spread of MRSA may comprise diverse measures in community and in healthcare. Effective means of preventing MRSA in community include strict antibiotic policy [38, 39] and increasing the awareness of MRSA in the general population, immigrants and livestock farmers. The global conundrum of antimicrobial resistance necessitates continuous surveillance of the MRSA situation as well as joint preventive actions also in countries with seemingly low MRSA incidence rates. The identification of MRSA reservoirs in the community is of utmost importance to protect hospitals from MRSA colonisation. Careful hand hygiene, barrier precautions in the care of MRSA carriers and screening of contacts are essential elements in preventing MRSA outbreaks in hospitals. Preadmission identification of MRSA risk factors and targeted screening can help to prevent the introduction of MRSA in hospitals.
